# Optimizing cell viability in droplet-based cell deposition

**DOI:** 10.1038/srep11304

**Published:** 2015-06-11

**Authors:** Jan Hendriks, Claas Willem Visser, Sieger Henke, Jeroen Leijten, Daniël B.F. Saris, Chao Sun, Detlef Lohse, Marcel Karperien

**Affiliations:** 1 Department of Developmental BioEngineering, MIRA institute for Biomedical Technology & Technical Medicine, Faculty of Science and Technology, University of Twente, The Netherlands; 2 Physics of Fluids Group, MIRA institute for Biomedical Technology & Technical Medicine, Faculty of Science and Technology, J. M. Burgers Centre for Fluid Dynamics, University of Twente, The Netherlands; 3 Department of Orthopedics, UMC Utrecht, The Netherlands; 4 Department of Reconstructive Medicine, MIRA institute for Biomedical Technology & Technical Medicine, Faculty of Science and Technology, University of Twente, The Netherlands

## Abstract

Biofabrication commonly involves the use of liquid droplets to transport cells to the printed structure. However, the viability of the cells after impact is poorly controlled and understood, hampering applications including cell spraying, inkjet bioprinting, and laser-assisted cell transfer. Here, we present an analytical model describing the cell viability after impact as a function of the cell-surrounding droplet characteristics. The model connects (1) the cell survival as a function of cell membrane elongation, (2) the membrane elongation as a function of the cell-containing droplet size and velocity, and (3) the substrate properties. The model is validated by cell viability measurements in cell spraying, which is a method for biofabrication and used for the treatment of burn wounds. The results allow for rational optimization of any droplet-based cell deposition technology, and we include practical suggestions to improve the cell viability in cell spraying.

Droplet-based cell deposition is receiving increasing attention as a tool to construct or fill a variety of biological tissues. Striking examples are the cell spray treatment of burns^1^,^2^ or ulcers^3^, which provide faster and improved healing and are currently introduced in clinical practice. With one successful application in place, we seek to expand to other clinical areas including laparoscopic, endoscopic, and arthroscopic procedures^4^. This opens the possibility for minimally invasive cell therapy for tissue regeneration. A second example is the fabrication of functional tissue replacements in a laboratory, to enable curing non-functional tissues^5^,^6^,^7^. In current biofabrication technologies including ink-jet bioprinting^8^,^9^,^10^, laser-induced forward transfer^11^, valve-based bioprinting^12^,^13^, and cell spraying^2^,^14^,^15^,^16^,^17^, the cell transport from the initial cell suspension, called “bio-ink”, to the manufactured tissue is achieved by liquid droplet ejection and deposition. Although these technologies allow for high-viability cell deposition, limited throughput, limited precision, and poorly optimized cell-containing bio-inks are major obstacles in the controlled deposition of cells, such as required for the fabrication of functional tissues^5^,^7^.

To solve these issues and thereby optimize droplet-based cell deposition, knowledge of the cell viability as a function of the cell-containing droplet size and impact velocity is crucial. Ideally, single, highly reproducible impacts of droplets containing a single cell would be monitored for a large range of the impact parameters (droplet size, velocity, and material properties). Drop-on-demand systems provide such highly reproducible droplets, but usually the impact parameter space is relatively narrow for the cell-containing liquids used^18^,^19^,^20^,^21^,^22^,^23^. Therefore, to study post-impact cell viability, we use cell spray deposition, which allows for a much larger range of impact parameters. The substantial influence of the spray parameters on post-impact cell viability^2^,^15^,^16^,^17^,^24^ suggests that cell viability can be controlled, providing a model system to assess cell survival after impact. Additionally, the shear stress exerted on the cell within the spray nozzle is much lower than the shear stress during impact, which allows for assessment of the impact process alone (for other technologies this is not the case, as explained in supplementary section I).

The current work aims to understand the influence of the droplet impact on cell viability, which is applicable both to drop-on-demand and spray deposition technologies. We introduce a model describing the cell viability as a function of the cell-containing droplet size, the viscosity, and the impact velocity. The model is validated by cell spray experiments, following a two-step approach. First, the droplet size and impact velocity are measured and used to obtain model predictions as described. Subsequently, the cell viability after spraying is measured as a function of the air pressure, the liquid viscosity, the nozzle-substrate distance, and the substrate stiffness. The model is shown to accurately describe the viability measurements as a function of the input parameters. These results provide a powerful tool to rationally evaluate and improve clinical spray treatments and tissue engineering applications.

## Results

### Cell viability model

**Figure 1:**
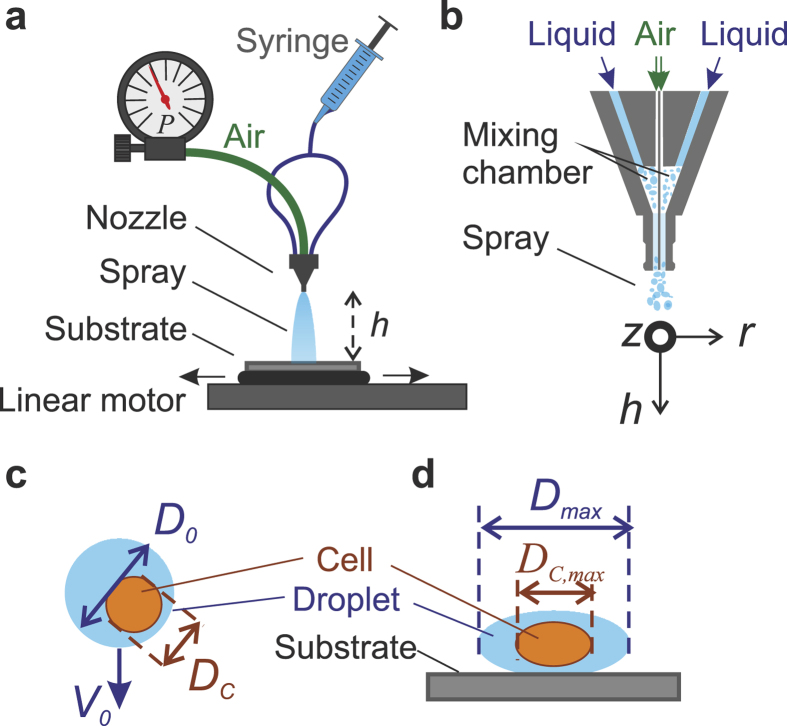
Overview of the experimental method and parameter definition. A two-phase spray nozzle is used to generate the spray, and placed at a distance *h* from the surface. The cell-containing liquid (with viscosity *μ*) is delivered to the nozzle using a syringe pump (not shown). Air at controlled pressure *P* is applied to the nozzle gas inlet. The impact substrate is moved under the spray (indicated by horizontal arrows) using a linear motor, ensuring clean and homogeneous impact. Generally, a clean glass substrate is used, but gelatin-water mixtures (with gelatin weight fractions *C*_*g*_) are used to assess the influence of the surface stiffness. (**b**) Cross-section of the nozzle, illustrating the air and liquid flows, and the coordinate system used. Figures (**c**) and (**d**) illustrate key variables describing the cell-containing droplets in air (**c**) and during impact (**d**).

In cell spraying, cell damage is primarily expected during impact of the cell-containing droplets (see supplementary section I). In particular, impact generally results in cell deformation and elongation of the cell membrane^25^, as illustrated in Fig. 1(c,d). For an increase of the cell membrane area up to ~5%, the membrane is stretched, but remains intact. However, for larger extensions, rupture can be observed^26^. As rupture generally results in cell death, the probability of survival *η* is modeled as a function of the relative cell membrane area *γ* (compared to the undisturbed case) according to ref. 27:


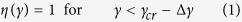



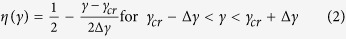



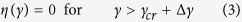


with *γ*_*cr*_ = 1.5 the critical membrane expansion as quantitatively provided in ref. 27, and 2Δ*γ* = 1 the range of surface expansion in which the cells partly survive. Key model conditions include an elastic cell response to stresses (which is fulfilled for shear rates 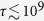
s^−1^^26^; for the estimate of the shear stress see supplementary section I) and negligible lipid membrane replenishment during deformation (fulfilled for 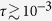 s^−1^^28^), which are met in the current work.

To obtain the relative cell membrane area *γ*, first a “clean” cell impact on a hard substrate is considered. The cell is described as a spherical liquid droplet with diameter *D*_*c*_, velocity *V*_*c*_, viscosity *μ*_*c*_ = 12 mPa s, density *ρ*_*c*_ = 1015 kg/m^3^ and surface tension *σ*_*c*_ = 0.072 N/m^2^ (key parameters are visualized in Fig. 1(c,d)). The maximal spreading diameter reached during impact *D*_*c,max,*0_ is then calculated as a function of the cell Weber number, which describes the ratio between kinetic energy and surface energy^29^,^30^,^31^:


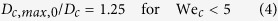



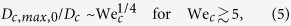


with cell Weber number 
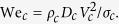
 These equations are valid for Newtonian liquids and for 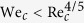
29 (which is fulfilled for our experiments), in which the cell Reynolds number Re_*c*_ = *ρ*_*c*_*V*_*c*_*D*_*c*_/*μ*_*c*_ represents the ratio between inertia and viscosity. The cell shape is defined by assuming cell deformation into an oblate spheroid and volume conservation. At the instant of reaching its maximal extension, its height equals 
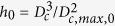
. This provides the deformation *M*_0_ for an impacting cell, which is defined according to ref. 25:


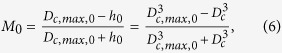


i.e. *M*_0_ = 0 for a sphere, and *M*_0_ = 1 for a plane.

The potentially large influence of the surrounding droplet on the cell’s deformation was modeled numerically by Tasoglu *et al.*^25^. Re-interpreting their results provides a quantitative expression capturing the cell deformation *M* as a function of *M*_0_, the surrounding droplet’s diameter *D*_0_, and its viscosity *μ*_0_ (for details see supplementary section III, for viscosity-dominated deformation of compound drops see ref. 32):


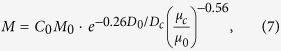


with *C*_0_ a fitting parameter (which is set to *C*_0_ = 5, as discussed in supplementary section III). Subsequently, *M* is translated into the maximal spreading diameter of the (oblate-spheroid) cell as 
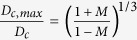
, which is used below to calculate the cell surface area. Since this occasionally results in a cell diameter exceeding the droplet’s diameter, we additionally implement the condition *D*_*c,max*_ = min(*D*_*c,max,*0_, *D*_*max*_). The surface area *A* of an oblate spheroid (the assumed shape of a single trypsinized cell) can be calculated as:


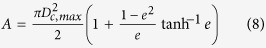


with 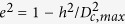. The relative surface area is given by 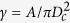, which completes the system.

Finally, the model is extended to account for the stiffness of the impact substrate. We assume impact on a liquid pool (with material properties equal to the droplet) as a soft surface limit. A droplet with diameter *D*_0_ and velocity *V*_0_ impacting on such a pool is (in first approximation) described by the impact of a droplet with diameter 2*D*_0_ and velocity 0.5*V*_0_ on a hard substrate^33^. As soft-surface droplet impact is not adequately understood even for basic model systems^34^, we propose an effective droplet diameter and velocity as:


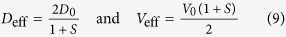


with *S* an arbitrary stiffness parameter ranging from *S* = 0 for liquid surfaces to *S* = 1 for hard surfaces. A gelatin-water mixture is used to generate a substrate stiffness range corresponding to a large variety of natural tissues^35^,^36^. In the high-shear regime associated to fast micro-droplet impact the viscoelastic properties of these substrates cannot be measured by any standard viscometer. Therefore, the gelatin mass fraction *C*_*g*_ is used to define the stiffness: *S* = *C*_1_*C*_*g*_, with *C*_1_ a fitting constant. Using *C*_1_ = 5 provides reasonable agreement between the model and our soft-surface impact measurements. For *C*_*g*_ > 1/*C*_1_ we define *S* = 1, which implies an effectively stiff surface for *C*_*g*_ > 0.2.

**Figure 2:**
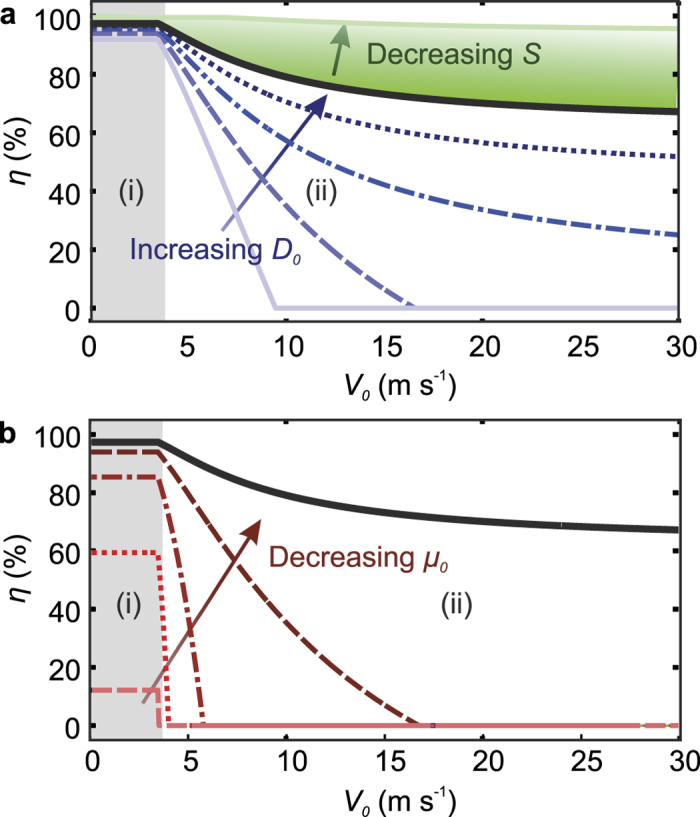
Model predictions of the post-impact cell survival probability *η* as a function of the impact velocity *V*_0_, for a single-cell containing droplet. Figure (**a**) shows the influence of the droplet diameter *D*_0_ (indicated by lines representing 1 ≤ *D*_0_/*D*_*c*_ ≤ 3 in steps of 0.5) and the surface stiffness (indicated by the color gradient representing *S* = 1 (stiff substrate) to *S* = 0 (liquid pool)). Figure (**b**) shows the influence of the droplet viscosity (lines plotter for *μ*_0_ = 1, 2, 4, 8, 12  mPa s). The solid black lines indicate the viability values obtained for the reference parameters: *D*_0_/*D*_*c*_ = 3, *μ*_*c*_/*μ*_0_ = 10, and *S* = 1. Region (i) (shaded) indicates the low-Weber number regime (We < 5). Here the cell deformation is small and independent of the impact velocity. In region (ii), decreasing viability is obtained for increasing velocities, due to increasing cell deformation.

Figure 2 shows example model results. The viability probability of individual cells is shown as a function of the impact velocity, for different sizes of the surrounding droplet (Fig. 2(a)), and different relative viscosities (Fig. 2(b)). At low velocities, the cell viability is only weakly dependent on the impact parameters since a small and constant cell deformation is assumed for low Weber numbers (We < 5). For increasing velocities (corresponding to We > 5) a decrease in cell viability is observed. In this regime, the size of the surrounding droplet and its viscosity strongly affect cell viability. Larger surrounding droplets provide stronger cushioning and thereby increase the viability (Fig. 2(a)). Increasing the droplet viscosity negatively influences the cell viability, since for *μ*_*c*_ = *μ*_0_ the droplet will flow around the (relatively stiff) cell, whereas for *μ*_*c*_ < *μ*_0_ the cell flows to dampen the (relatively stiff) droplet’s impact, resulting in significant cell deformation and decreased viability. Finally, softer substrates provide increased cushioning as shown by the color gradient in Fig. 2(a). Here, the surface deforms such that the deformation of the droplet is reduced. Consequently, cell deformation is suppressed and a higher viability is expected. In conclusion, optimal cell viability is expected for slow, large, and low-viscosity cell-containing droplets impacting onto a soft surface.

### Spray characterization

**Figure 3:**
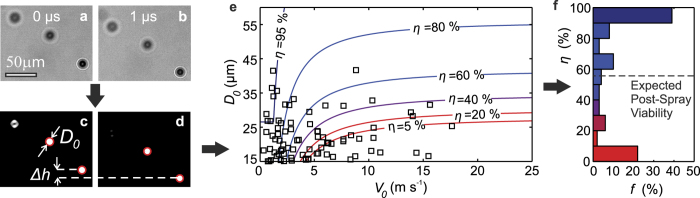
Characterization of the spray. An example image pair is shown before ((**a**) and (**b**)) and after image processing ((**c**) and (**d**)). As indicated by the circles in (**c**) and (**d**), two droplets are automatically detected using home-written image analysis software, which provides their diameter *D*_0_ and translation Δ*h*. Figure (**e**) shows the droplet size- and velocity for *P* = 0.4 .10^5^ Pa, *μ* = 1 mPa s, *h* = 3 cm. The markers (◻) show 100 statistically representative droplets constituting the spray. The contours represent predicted cell viability values (*η*). Figure (**f**) shows the relative incidence *f* of each cell survival probability *η*, which is binned for the sake of clarity. Averaging these probability values results in the expected post-spray cell viability (dashed line). The expected cell viability strongly depends on the spray parameters setting, as plotted in Fig. 5 (red diamond).

To obtain cell viability predictions from the model, the droplet size- and velocity are required. We obtain these parameters according to Fig. 3. First, the droplets are visualized as shown in Fig. 3(a,b). Automated image analysis then provides the droplet diameter *D*_0_ and velocity *V*_0_ = Δ*h*/Δ*t*, as illustrated in Fig. 3(c,d). For any spray experiment, a wide range of droplet sizes and velocities is observed. A representative sample of droplet sizes and velocities is indicated by the black markers in Fig. 3(e), which also contains indicative cell viability contours (similar graphs for different spraying parameters are included in supplementary section II). To obtain the cell viability, for each drop the cell survival probability was calculated as a function of the size, speed, viscosity, and surface stiffness. This provides a distribution of cell viability probabilities as shown in Fig. 3(f) (corresponding to the drop size and speed distributions), of which the average provides a single predicted cell viability value which is compared to experimental viability data in the following section.

### Cell viability measurements and model validation

**Figure 4:**
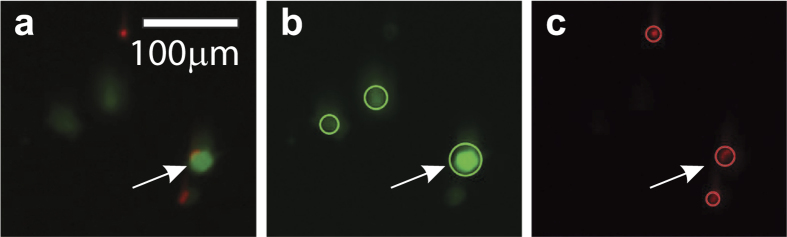
Example live-dead assay. Figure (**a**) shows the original image; figure (**b**) shows the calcein staining (in green), and figure (**c**) shows the EthD staining (in red). The circles in figures (**b**) and (**c**) indicate the automatically detected cells for each staining. The arrows show a cell in which both stainings are retrieved. These cells are considered damaged but viable, and therefore counted as live.

**Figure 5:**
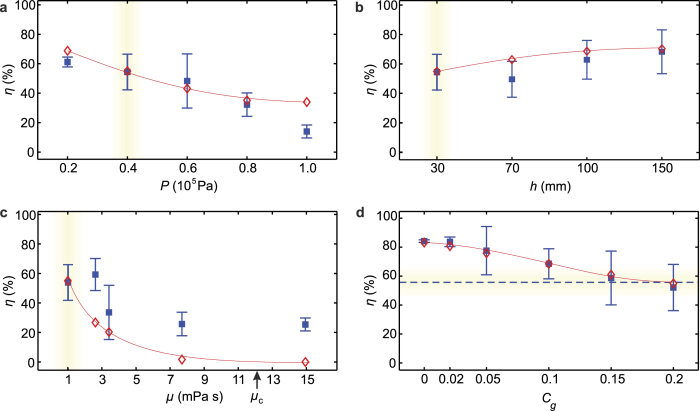
Cell viability *η* as a function of the spray control parameters. Figures (**a**–**d**) indicate the viability as a function of the pressure *P*, the nozzle-substrate distance *h*, the liquid viscosity *μ*, and the substrate gelatin percentage, respectively. Measured values are indicated as blue squares 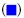 with the error bar representing the standard deviation; red diamonds 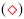 indicate model predictions. The lines are a guide to the eye for the model predictions (which cannot be displayed as smooth curves as they depend on the spray distributions, as explained in supplementary section II). The reference settings are *P* = 0.4 .10^5^ Pa, *μ* = 1 mPa s, *h* = 3 cm, and a glass impact substrate, as indicated by the yellow shade in each plot.

To measure the post-impact cell viability, spray experiments are performed using the setup displayed in Fig. 1. After spraying, the cells are collected and stained in a live-dead assay of which an example is shown in Fig. 4. Using automated image analysis, the cell viability is determined for each measurement. Figure 5 shows the measured cell viability as a function of the pressure, the nozzle-substrate distance, the viscosity, and the surface stiffness. The cell viability is also measured as a function of the time after spraying (see supplementary section V), demonstrating the long-term viability of the surviving cells.

**Figure 6:**
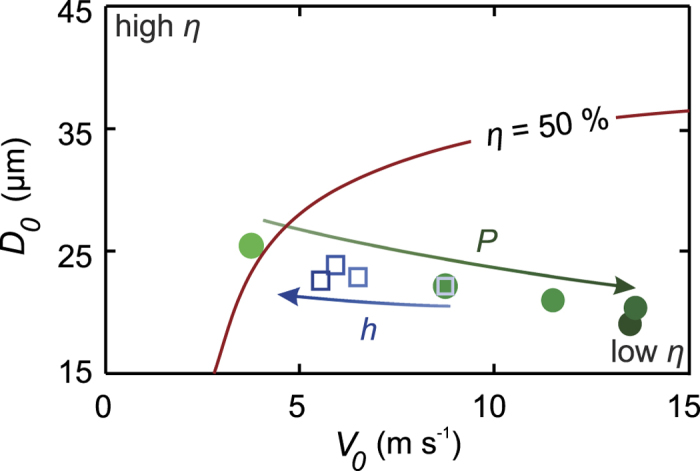
Viability as a function of the droplet size and speed. The line represents the 50% viability contour; additional contours are plotted in Fig. 3(e). The dots indicate the mean diameter versus the mean velocity as a function of the spray pressure (the arrow indicates increasing pressure for *P* = [0.2, 0.4, 0.6, 0.8, 1].10^5^ Pa). The open squares indicate the distance from the nozzle for *h* = [30, 50, 100, 150] mm. For increasing pressure, the droplet size decreases and the impact velocity increases, resulting in a lower viability. For a larger distance from the nozzle, the impact speed decreases, resulting in improved viability. The influence of the viscosity and the surface stiffness is displayed in Fig. 2), since these variables do not affect the droplet size and speed.

The model is compared to cell viability measurements in Fig. 5. Good agreement is observed for the pressure, the nozzle-substrate distance, and the surface stiffness. In particular, except for the lower and upper pressure data points, the measured data is quantitatively described by the model, which is remarkable in view of the single fitting parameter used. Our model over-estimates the influence of increased viscosity, but still captures the trend. The origin of the lower cell viability for increasing pressure (4(a)) is that the spray consists of smaller drops impacting at increasing velocities, as shown in Fig. 6. Both factors result in stronger cell deformation and therefore cell death. Increasing the nozzle-substrate distance improves the cell viability, as shown in Fig. 5(b). Figure 6 shows that this trend is primarily caused by the decreasing droplet velocity far from the nozzle, which results in less deformation of the cell and increased viability. Higher viscosities result in a lower cell viability, as shown in Fig. 5(c). For high viscosity, the deformation of the cell-containing droplet primarily occurs within the cell. Consequently, the cell membrane is significantly stretched, and the viability decreases. Finally, decreasing the surface stiffness improves the viability (Fig. 5(d)), since the deforming surface “cushions” the impacting cell-containing droplet.

## Discussion

The model assumptions affect its applicability, and therefore deserve further discussion. First, the assumption that the cells are centered in the droplet will be approximately true for drop sizes just exceeding the cell size, but for larger droplets the cells may be located at the edge of the spreading film where the shear stresses are much higher than in the center^31^. Therefore, for larger drops such as generated for low pressures (see supplementary Figure 4(a)), the cell viability may be suppressed. This mechanism possibly explains the lower viability for *P* = 0.2 .10^5^ Pa observed in Fig. 5(a). Second, following ref. 25, we assume that the cell viscosity is independent of the shear stress. As experiments reveal shear-thinning behavior of cells^37^,^38^, implementing shear thinning could further improve the model. Third, the influence of the droplet volume on the number of cells per drop is not yet taken into account. Finally, the influence of viscosity on cell deformation might be different from equation (7), since equation (7) is derived from numerical results for much larger cell-containing droplets (ref. 25, also see supplementary section III) whereas most of our droplets have a size just exceeding the cell size (supplementary Figure 3). Avoiding these assumptions by extending the model may result in even better agreement. Still, the agreement between our model and measurements is remarkable and provides evidence for correctly capturing the underlying physics.

Droplet impact-induced cell damage has far-reaching consequences for users as well as developers of cell spraying and other bioprinting technologies. In clinical practice, the need for adequate cell-spraying protocols^39^ is even more pressing than expected. In particular, manual operation of spray devices is common practice, but associated with variations in nozzle-substrate distance, the air pressure, and the viscosity. These variables should be carefully controlled to ensure high cell viabilities. The actual values may still depend on the nozzle design and the cell type, but increasing the spray distance and using low-viscosity spray suspensions, while avoiding hard impact surfaces, will generally improve cell survival.

However, harmful spraying conditions cannot always be avoided due to treatment-specific clinical requirements or constraints in biofabrication. For example, in arthroscopic procedures, the nozzle-surface distance is limited to at most 1 cm^4^. In view of our results, it is unclear whether the treatment success shown for burn treatments, where this distance usually exceeds 10 cm, can be reproduced in arthroscopic application (see Fig. 5(b)). Also, in many treatments, the impact surface is a tissue defect and therefore cannot be freely chosen or altered. Our hardest gelatin-containing surfaces result in similarly low viability as hard glass surfaces. These 20% gelatin surfaces are similar in stiffness to muscle tissue^35^, which is one of the softer human tissues^36^. Thus, clinically relevant surfaces are relatively stiff, possibly affecting cell survival. To solve this problem, the spray parameters require optimization. Similarly, the deposition of viscous, cell-containing hydrogels is usually required to preserve the desired 3D tissue architecture in biofabrication^12^. However, such liquids are likely to negatively affect the cell viability (see Fig. 5(c)). Decreasing the spray pressure^2^,^15^,^16^,^17^, using softer impact surfaces^24^, or increasing the nozzle-substrate distance can counteract the negative influence of the increased viscosity (see Fig. 5), but, unfortunately, these parameters are sometimes also constrained.

The greatest potential to improve cell viability in cell spraying therefore seems to be optimization of spray nozzle designs. Particularly, design optimization resulting in increased and monodisperse droplet sizes and reduced impact velocities would allow for successful cell deposition in an extended viscosity range. In this study we have used the Duploject system, which is approved for clinical application of fibrin glue by spraying and also used for cell spraying^4^. The spray produced by this nozzle is characterized by highly polydisperse droplet diameters and velocities. High-velocity impacts occur even for the most gentle spraying parameters, limiting the measured highest post-spray cell viabilities to 90%. Nozzle designs producing more monodisperse droplets may prevent these lethal events, even for high-throughput spraying of viscous liquids. Our experimental setup can be used to rationally optimize such future spray nozzles, which may substantially enhance the application window of cell-spraying.

Finally, other droplet-based cell deposition technologies may benefit from our approach. In ink-jet bioprinting, highly monodisperse cell-containing droplets^40^,^41^,^42^,^43^ are deposited. Here, typically, low-viscosity droplets of 40 m (exceeding the cell size by a factor of 3) impact at velocities below 10 ms^−1^. Good viability is generally measured in this range^23^, in agreement with our model (Fig. 2). However, reduced cell viability is observed for neural cells^44^. Our study suggests that especially decreasing the liquid viscosity or reducing the droplet ejection velocity could improve the cell viability of such more fragile cell types. These measures reduce the expected cell damage both in the nozzle and during impact, which are both likely causes of cell damage in ink-jet printing (see supplementary section 1). Using soft impact substrates or larger nozzle-substrate distances will still reduce impact-related damage, but nozzle-induced cell damage or poration^10^ cannot be suppressed in this manner.

Cell viability trends observed in laser-assisted bioprinting (LAB) are also described by our model. Here, a pulsed laser is focused onto a cell-containing liquid film, resulting in the deformation of this film and break-up into cell-containing droplets^45^,^46^. By placing a receiver substrate in the line-of-flight of the cell-containing droplet, deposition is achieved. Increased impact velocities result in decreased cell viability^47^,^48^, and soft impact surfaces improve cell survival^49^, analogous to our model results shown in Fig. 2. Surprisingly, improved cell viability was reported for increased viscosity^48^, but this was explained by the reduced impact velocity due to the increased viscosity. Studying the cell viability as a function of the liquid viscosity at a controlled impact velocity would therefore be highly interesting. Such experiments may also advance the understanding of cell membrane deformation due to pulsed shear stresses^50^, as occurring in cell-containing droplet impact.

In conclusion, we present and validate an analytical model describing the cell viability as a function of the droplet impact parameters. The model describes cell-viability trends in cell spraying, inkjet bioprinting, and laser-assisted cell transfer, confirming the general importance of droplet impact for cell survival in bioprinting. Since future biofabrication applications may involve high-throughput deposition of different, possibly more fragile, cell types contained by high-viscosity bio-inks, we expect that preventing cell damage will become even more important. In particular, post-spray cell survival will be cell-type dependent. In addition, different bio-inks may require distinct deposition parameters in combination with dedicated nozzle designs allowing monodisperse droplet ejection while maintaining low shear rates inside the nozzle. Our study provides a framework to optimize cell survival in such future applications, contributing to reliable biofabrication of complex 3D-tissue constructs of a clinically relevant size.

## Methods

### Cell culture

Neonatal rat dermal fibroblasts (ITK Diagnostics) were cultured in Minimum Essential Medium α (α-MEM) (Gibco) supplemented with 10% Fetal Bovine Serum (FBS) (Lonza), 1% L-Glutamine (Gibco) and 1% Pen/Strep (Gibco) at 37 °C and 5% CO_2_.

### Cell suspension

Cells were harvested at 80% confluence by trypsinization and suspended at 1.5 × 10^6^ cells per ml in culture medium excluding FBS for impact experiments. Optionally dextran (Sigma, 15–25 kDa) was added to the cell suspensions to increase the viscosity. The influence of the dextran concentration on the liquid viscosity was measured using a viscometer (Rheolab QC, Anton Paar). As shown in supplementary Figure 10, the measured viscosity is in agreement with literature values.

### Cell viability measurements

Cell spray experiments were performed with the set-up shown in Fig. 1. Using a syringe pump, the cell suspension was pushed through a spray nozzle (Duploject spray system (Baxter AG). Photographs of this system, which is also known as bio-airbrush, are provided in supplementary Figure 9) at a controlled liquid flow rate of 2.4 ml per min. The impact surface consisted of a standard clean microscope slide covered with a PDMS mask to ensure a defined and reproducible impact area. These slides were optionally coated with a layer of gelatin (Type A, Sigma, *d* = 0.5 mm), in order to adjust the surface stiffness. A crucial aspect of the experiment is that impact occurs on a dry surface, i.e. that the cell spray does not impact onto previously sprayed droplets. To ensure this, the impact surface was reproducibly moved using a programmable linear motor. The substrate velocity was set such that 12 ± 0.5 mg of the sprayed liquid was collected for each experiment (the velocity was decreased for increasing nozzle-substrate distances since the spray density decreases for increasing distance from the nozzle). This weight corresponds to a liquid film of 18 μm (which is equivalent to ~1 cell thickness) and resulted in covering roughly half of the surface area with sprayed droplets, such that most droplets land on the dry surface. Within 10 s after each spray experiment, the surface was rinsed with FBS-free culture medium to collect the cells. The cells rinsed from three different samples (sprayed using equal parameter settings) were collected into a 12 ml centrifuge tube (Greiner). The contents of each tube were subsequently processed in a live-dead assay. For each parameter setting, three independent spray cycles were performed (i.e. 9 impact surfaces were collected in three different tubes). This approach provided three data points for each parameter setting, which were used to display the error bar in Fig. 5.

### Live-dead assay

The collected cells were incubated in phosphate buffered saline (PBS), supplemented with 1 nM calcein AM and 6 nM ethidium homodimer, at 37 °C and 5% CO_2_ for 30 minutes. Each stained cell suspension sample was transferred to a well in a 24 wells-plate and 8 random spots per well were imaged (EVOS Fl microscope), within 2 hours after each spray experiment. Live (green) and dead (red) cells were automatically counted using a home-written Matlab script (Fig. 5 shows an example), resulting in an average of 450 ± 332 (s.d.) detected cells per sample (with a minimum of 12 cells). Cells stained both live and dead were considered damaged, but viable, so counted as alive. For each sample, viability was calculated accordingly. All measurements were subsequently corrected by setting the control viability (non-impact, measured at the same time) to 100%.

### Spray characterization

As cell survival critically depends on the characteristics of the cell-containing droplets, the spray characteristics were determined in detail. The setup used is shown in supplementary Fig. 2. All spray-generating components were equal to the components used for assessment of the cell viability (Fig. 1). A dual-pulse ND:Yag laser with a pulse duration of 6 ns was used for brightfield illumination. To prevent fringes, the coherent laser light was diffused using a fluorescent plate placed in front of the laser. The non-coherent pulses were captured by a dual-shutter camera (Sensicam, PCO). The time delay between the illumination pulses was set to 1 μs, which is sufficiently long to measure the translation Δ*h* of the droplets while preventing confusion between different droplets. All timings were controlled using a BNC 575 pulse-delay generator (not shown). A 10× long-distance objective was used, resulting in a field of view of 0.67 × 0.89 mm2. As the focal plane thickness of this objective is *δ*_*F*_ ≈ 0.1 ± 0.03 mm, a volume of 0.67 × 0.89 × 0.1 mm3 was visualized. As the spray is much larger than this volume, measurements were taken at different *z*- and *h*-positions to fully characterize the spray, as illustrated by the small rectangles in supplementary Figure 1(b) (the *r*-position is maintained at the nozzle axis).

For each measurement, 400 image pairs were obtained. Example images are shown in Fig. 3(a,b), where the downward translation of the droplets is clearly visible. Motion blur was prevented by the short illumination pulses of 6 ns. The droplets were automatically detected using a home-written Matlab script. Droplets appearing sufficiently sharp were automatically selected, as illustrated by the red circles in Fig. 3(c,d). Here, the top-left droplet was too blurred for detection and discarded. Although the droplets in Fig. 3 are similar in size, the image processing software allowed for successful detection of droplets in the range of 1 ≤ *D*_0_ ≤ 100 μm.

### Soft substrate preparation

Glass microscope slides were optionally coated with a layer of gelatin (Type A, Sigma) of thickness 0.5 mm, and kept in air at room temperature for 30 min prior to the experiment. The surface stiffness was adjusted by adding gelatin mass fractions of *C*_*g*_ = [2, 5, 10, 20]% to PBS. PBS without gelatin was used in the soft-surface limit.

## Additional Information

**How to cite this article**: Hendriks, J. *et al.* Optimizing cell viability in droplet-based cell deposition. *Sci. Rep.* **5**, 11304; doi: 10.1038/srep11304 (2015).

## Supplementary Material

Supplementary Information
