# Supplementary material for: Optimizing cell viability in droplet-based cell deposition
Source: Sci Rep. 2015 Jun 11;5:11304. doi: 10.1038/srep11304 (PMC5387118; doi:10.1038/srep11304)
Supplement: Supplementary Information [file srep11304-s1.pdf]

Supplementary material of: Optimizing cell viability in droplet-based cell deposition

Jan Hendriks, Claas Willem Visser, Sieger Henke, Jeroen Leijten, Daniël B.F. Saris, Chao Sun, Detlef Lohse, Marcel Karperien

March 30, 2015

I. Shear rate analysis The shear rates in the spray nozzle and during impact are estimated. The shear rate is defined as $\gamma(h) = \partial u/\partial h$, with $u(r)$ the velocity in r-direction. The coordinate system shown in supplementary figure 1. By dimensional analysis the shear rate during impact is estimated as $\gamma \sim V_0/D_0 \approx 10^6$ s^{-1} for a $D_0 = 10$ µm droplet impacting at 10 ms^{-1}. For drop-on-demand systems (i.e. ink-jet printing, valve-based deposition, and certain regimes of laser-induced forward transfer) both the velocity inside the nozzle and the diameter of the nozzle are of the same order as D_0 and V_0, respectively. Therefore, distinguishing the nozzle-induced shear from the impact-induced shear is far from trivial. However, for the cell spray, the diameter of the nozzle $D_N \sim 500$ µm is much larger than D_0, resulting in a strongly reduced shear rate within the nozzle, which is estimated as $\gamma \sim V_0/D_N \approx 2 \cdot 10^4$ s^{-1}. Therefore, only the impact of cell-containing droplets is expected to have a significant shear-induced effect in cell spraying.

II. Spray characterization
As described in the Methods section, the spray is characterized using the setup shown in supplementary figure 1. Supplementary figure 2 shows images of the spray as a function of the air pressure similar to observation with the naked eye. The spray visually changes as a function of the pressure and widens with increasing distance from the nozzle. Some droplets are individually visible, but these only represent the and largest and slowest droplets.

Key spray characteristics are shown in supplementary figure 3. Supplementary figures 3 (a) and (b) respectively show that the droplet diameter decreases for increasing pressure, and that the droplet velocity increases for increasing pressures. Supplementary figure 3 (c) illustrates the strong influence of the nozzle-substrate distance on the spray velocity. In addition, we found that the nozzle-substrate distance hardly affects the droplet diameter, and that the viscosity neither affects the droplet diameter nor the droplet velocity (not shown).

The influence of the spray characteristics on the cell viability is shown in supplementary figures 4 to 7. Here, the droplet size- and velocity distributions are plotted for a range of spraying parameters (left figures). In each plot, the corresponding viability probability contours are also plotted. For increasing pressure, the droplet size decreases and the droplet velocity strongly increases, which results in more low-viability impacts as observed by comparing supplementary figure 4 (a) and (c). This trend is reflected in supplementary figure 4 (d), where almost half of the cells will certainly not survive the impact. Increasing the distance from the nozzle primarily decreases the impact

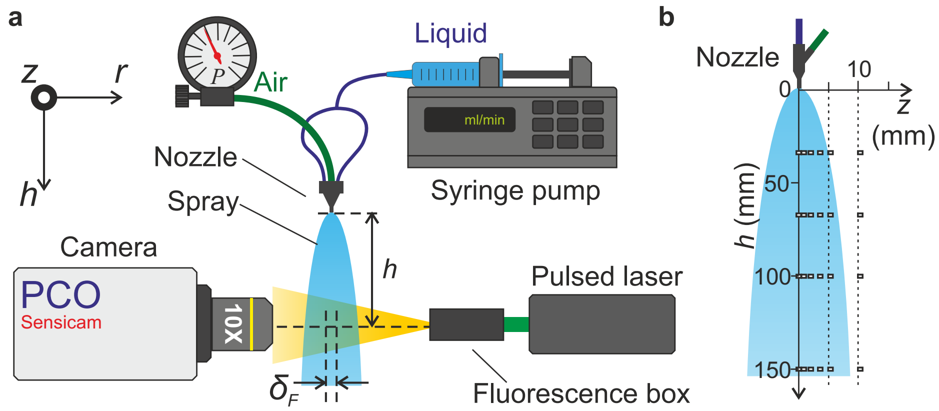

Supplementary figure 1: (a) Overview of the setup used for the spray characterization measurements. For visualization, a dual-shutter camera is used. For each image a slice of the spray is visualized, corresponding to the depth of the focal plane δ_F. Illumination is provided by dual-pulse ND:Yag laser combined with a fluorescence box to remove the laser coherence. The spray generation is equal to the cell spraying experiments. (b) Side-view of the spray. Each small rectangle indicates a measurement location used for spray characterization, corresponding to the field of view of the camera (drawn to scale) .

veloctity, as shown in supplementary figure 5 (a) and (c). Consequently, the viability increases as shown in supplementary figure 5 (d).

Increasing the viscosity hardly affects the droplet size- and velocity-distributions. However, for a given droplet size and velocity, the viability dramatically decreases, resulting in a strong shift of the viability contours shown in supplementary figure 6. Similarly, the viability contours change as a function of the substrate stiffness, resulting in increased viability for softer surfaces as shown in supplementary figure 7.

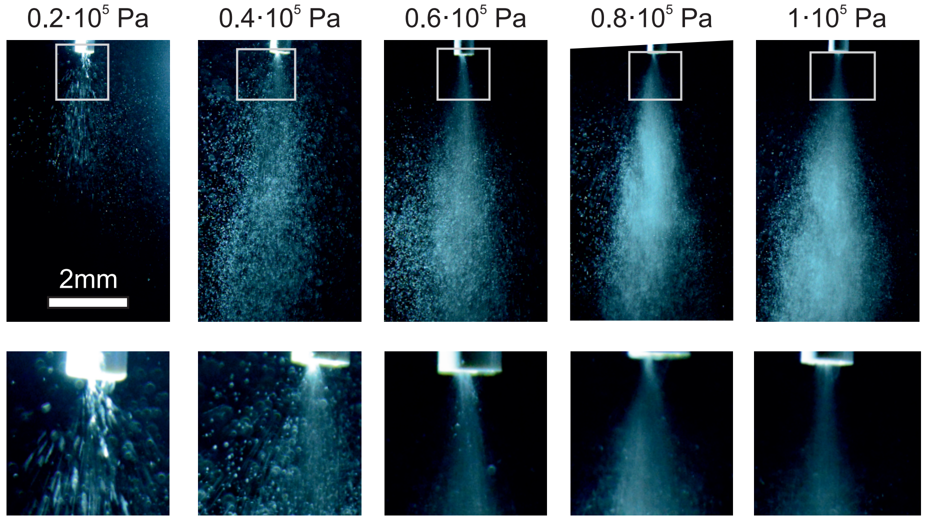

Supplementary figure 2: Low-speed images of the spray as a function of the air pressure. Each bottom images represents a magnification of the white rectangle in the top image. For $P = 0.2 \cdot 10^5$ Pa the droplets in the spray appear as stripes, due to motion blur. At higher pressures the individual droplets within the spray are no longer visible. The droplets which appear spherical fall through the surrounding air at very low velocity (i.e. they are not part of the main spray), and constitute a nebula around the spray.

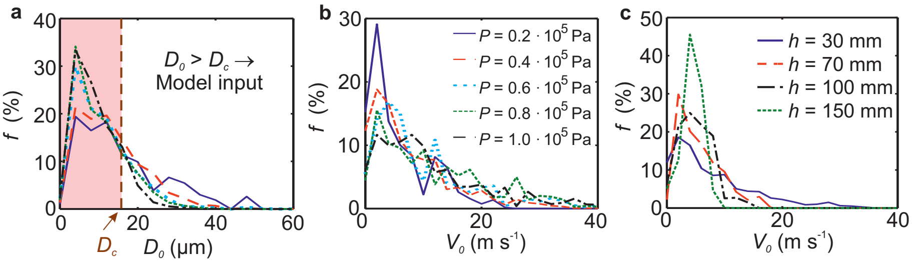

Supplementary figure 3: Figure (a) shows the spatially-averaged droplet diameter distribution for different air pressures (legend in figure (b)). The shaded area indicates droplet sizes D_0 smaller than the cell size $D_c = 15\mu\text{m}$, which are omitted in cell viability predictions. Figures (b) and (c) show the droplet velocity distributions for different air pressures (b) and distances from the nozzle (c), for droplets diameters exceeding the cell size. Unless specified in the legend, $P = 0.4 \cdot 10^5$ Pa, $\mu = 1$ mPa s, $h = 30$ mm, and $z = 0$ mm.

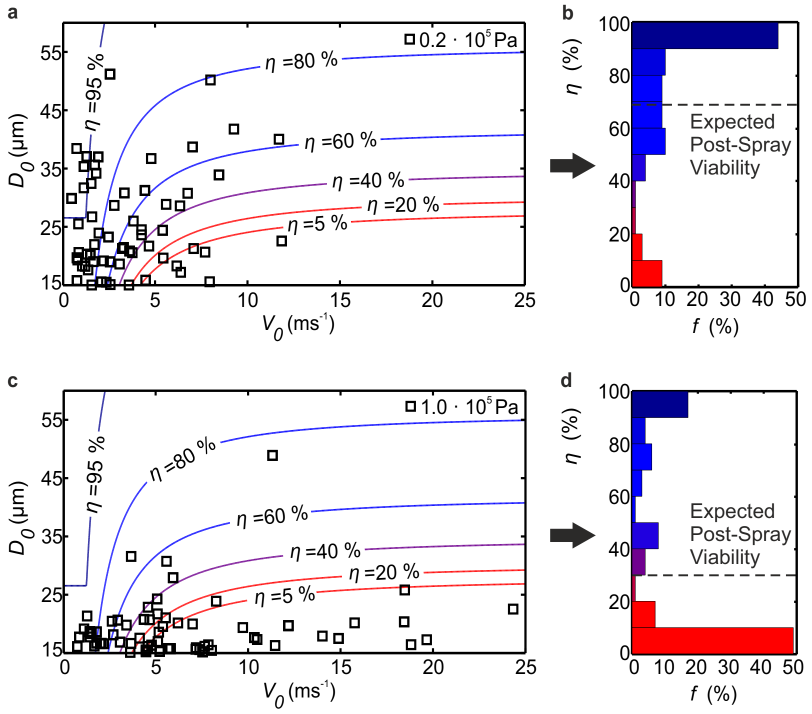

Supplementary figure 4: Droplet size- and velocity for a pressure $P = 0.2 \cdot 10^5$ Pa (a) and $P = 1 \cdot 10^5$ Pa (c). Representative cell viability contours are plotted in both figures. For $P = 1 \cdot 10^5$ Pa, more small and fast droplets are observed, which corresponds to low-viability regions of the diagram, and a lower predicted cell viability. Cell viability distributions are shown in figures (b) and (d), for pressure $P = 0.2 \cdot 10^5$ Pa (c) and $P = 1 \cdot 10^5$ Pa (d). The graphs are obtained analogous to figure 3(f) in the main text. The other control parameters are maintained constant at nozzle-substrate distance $h = 30$ mm, viscosity $\mu = 1$ mPa s, and a glass impact surface for which stiffness $S = 1$.

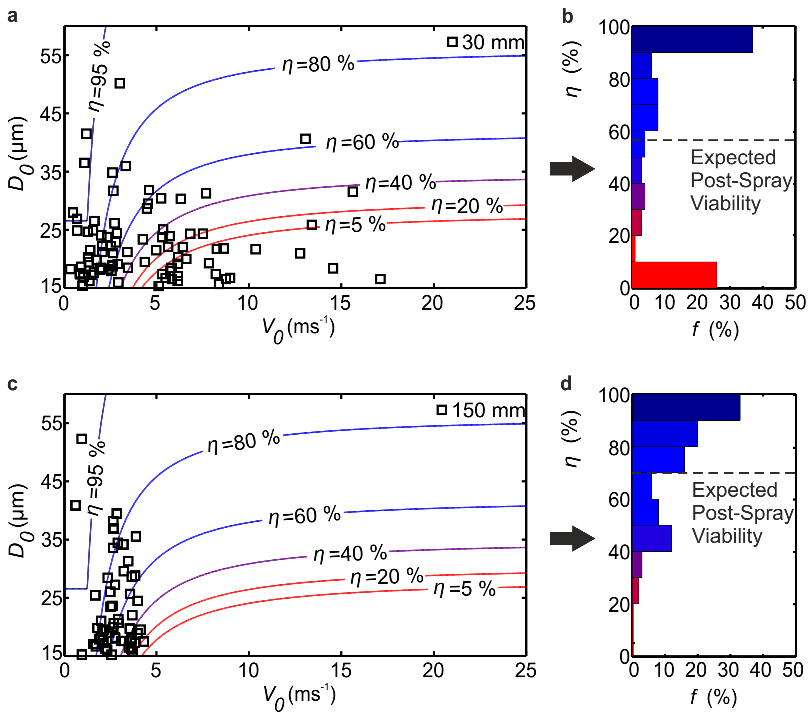

Supplementary figure 5: Droplet size- and velocity for a nozzle-substrate distance $h = 30$ mm (a) and $h = 150$ mm (c). For increasing distance, primarily the droplet velocity is decreased and the distribution corresponds to a high-viability region of the diagram. The increased viability is reflected in the cell viability distributions as shown in figures (b) and (d), respectively. The other control parameters are maintained constant at $P = 0.4 \cdot 10^5$ Pa, viscosity $\mu = 1$ mPa s, and a glass impact surface for which stiffness $S = 1$.

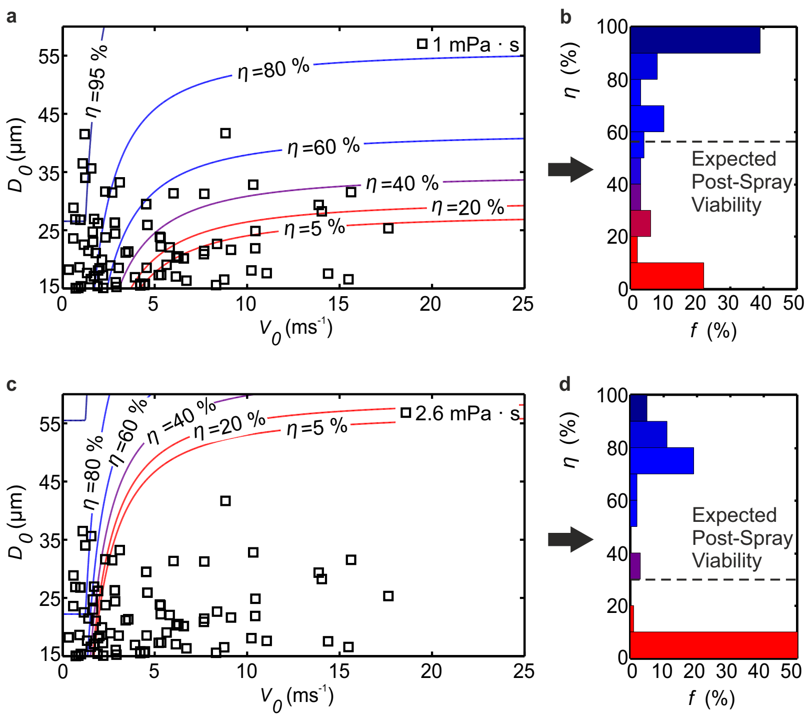

Supplementary figure 6: Droplet size- and velocity for a liquid viscosity$\mu = 1$ mPa s (a) and $\mu = 2.6$ mPa s (c). The increasing viscosity hardly affects the droplet size- and velocity distribution. However, the expected viability sharply drops for a constant droplet size and speed, which is reflected by a change in the viability contours in figure (c) as compared to (a). The reduced viability is reflected in the cell viability distributions as shown in figures (b) and (d), respectively. The other control parameters are maintained constant at pressure $P = 0.4{\cdot}10^5$ Pa, nozzle-substrate distance $h = 30$ mm, and a glass impact surface for which stiffness $S = 1$.

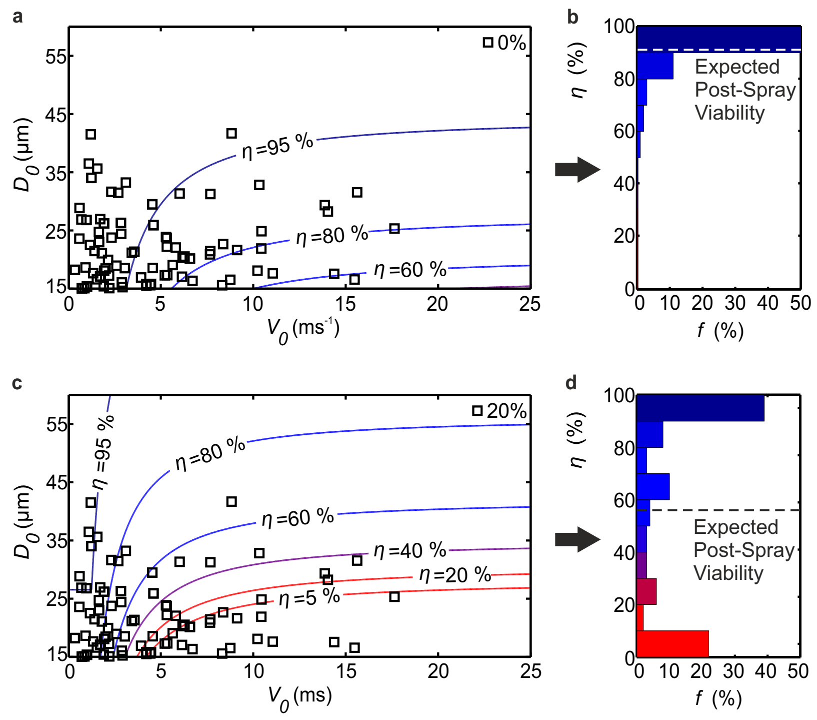

Supplementary figure 7: Influence of the substrate gelatin percentace: $C_g = 0\%$ in figure (a) (water surface, corresponding to stiffness $S = 0$) and $C_g = 20\%$ in figure (c) (corresponding to stiffness $S = 1$). The expected viability increases for a constant droplet size and speed, which is reflected by the difference between the viability contours in figure (c) as compared to (a). The increased viability is reflected in the cell viability distributions as shown in figures (b) and (d), respectively. The other control parameters are maintained constant at pressure $P = 0.4 \cdot 10^5$ Pa, nozzle-substrate distance $h = 30$ mm, and viscosity $\mu = 1$ mPa s.

III. Influence of the surrounding droplet on cell deformation

The influence of the cell diameter and impact velocity on cell deformation (equations (4) and (5) of the main text) are obtained from basic models describing droplet impact [1]. The influence of the surrounding droplet's diameter D_0 and viscosity μ on the cell deformation are obtained from the numerical results in ref. [2], as incorporated in equation (7) of the main text (the procedure is discussed below).

To obtain the influence of the surrounding droplet's diameter D_0 and viscosity μ on the cell deformation, the maximal cell deformation values provided in figures 15 and 19 in ref. [2] are extracted and plotted in supplementary figure 8 (a) and (b). A priori, the scaling of M as a function of these parameters is unknown. Therefore, different fit functions were tested and the best results are indicated by the dashed lines within these plots (the functional form is provided by the equations). Supplementary figure 8 (c) shows M as a function of both control parameters. Finally, supplementary figure 8 (d) shows the predicted increase in surface area as a function of the surrounding droplet's diameter D_0 and viscosity μ, using γ and equation (8) from the main text.

In our work, other relevant control parameters (e.g. surface tension and contact angle [2]) are not varied, and therefore captured by a constant prefactor C_0 in equation (7) of the main text. Fitting C_0 to our reference measurement (for which $P = 0.4 \cdot 10^5$ Pa, $\mu = 1$ mPa s, $h = 30$ mm, and a glass impact substrate) provides $C_0 = 5$. Importantly, even if the contact angle would be varied (for example by spraying onto a hydrophobic substrate), we would not expect a significant influence of this parameter, since the contact angle does not affect the spreading behavior of the drop for high Weber number impact [3]. Therefore, the influence of the contact angle on the outer drops spreading (and therefore cell deformation) is expected to be negligible. For low-velocity impact ($V_0 \lesssim 1$ m/s) the Weber number becomes of order 1. In this case, the contact angle drives the drops spreading, and is therefore expected to affect cell deformation resulting in a change of C_0.

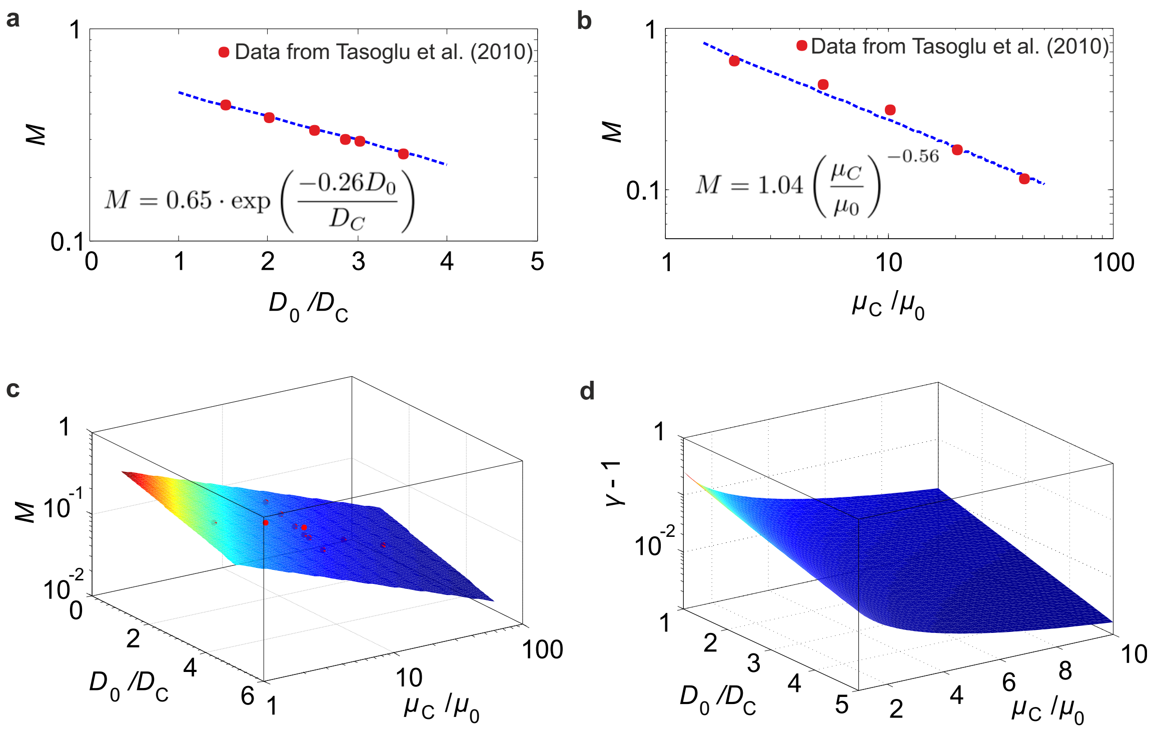

Supplementary figure 8: Cell deformation as a function of (a) the relative droplet diameter D_0/D_C and (b) the relative viscosity μ_C/μ_0. The dashed lines indicate our fit, as indicated by the equations within the plots. The data in figures (a) and (b) are the maximal values of the curves in figures 15 and 19 in Tasoglu *et al.*, respectively. (c) The cell deformation M and (d) the change in relative surface area $\gamma - 1 = A/\pi D_c - 1$ as a function of the droplet diameter and viscosity.

IV. Material characterization

See supplementary figure 9 for images of the spray nozzle, and supplementary figure 10 for the measured dextran viscosity.

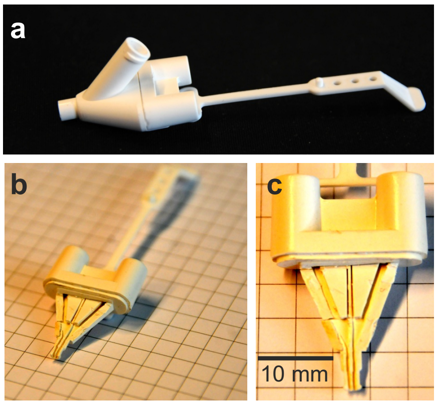

Supplementary figure 9: Images of the DuploJect nozzle used. (a) Overview image. The air enters through the top inlet, the liquid enters through the side inlets. (b) Nozzle that is cut to reveal the internal structure (the air inlet is also removed). (c) Top-view of the cut nozzle.

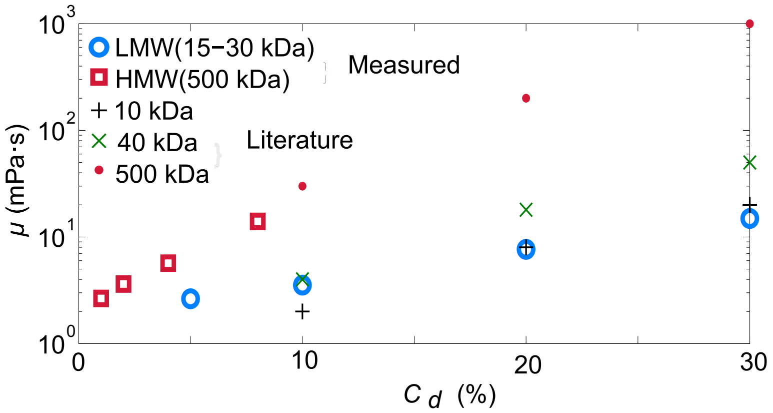

Supplementary figure 10: Measured viscosity as a function of the dextran concentration (mass %). Literature values [4] are shown for reference, indicating reasonable agreement.

V. Cell survival in time

The long-term viability of the cells is crucial both for clinical and laboratory application of cell spraying, and is plotted in supplementary figure 11a. All measurements are corrected by setting the control viability (non-impact, measured at the same time point) to 100%. Initially, after 2 hours, the control-corrected viability of 65% is comparable to the reference measurement. After 96 hours, the viability increases to around 90%. Although this shows that the cell population recovers after spraying, a considerable time is required before a high viability is reached. Spraying with high-viability parameters will prevent this problem, and may also reduce the potential influence of dead cells on the sprayed cell structure.

The absolute cell counts (normalized by the value after 2 hours) are plotted in supplementary figure 11b. The number of viable cells increases considerably in time, due to proliferation of surviving cells resulting in small cell clusters around these cells. This confirms the long-term viability and health of the surviving cells.

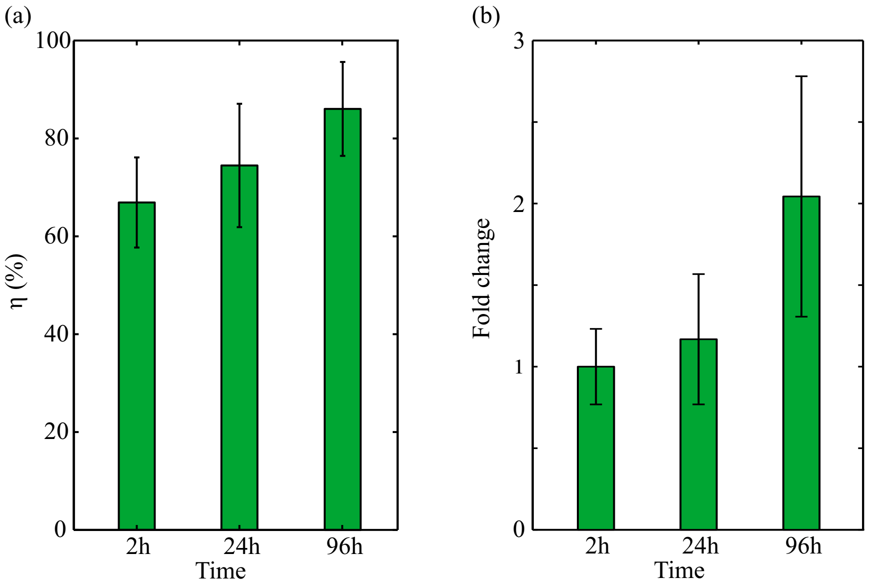

Supplementary figure 11: Cell survival and proliferation in time, for the standard experimental parameters ($P = 0.4 \cdot 10^5$ Pa, viscosity $\mu = 1$ mPa s, $h = 30$ mm, and a glass impact surface for which stiffness $S = 1$). Figure (a) shows that the initial viability of 65% steadily increases in time, to reach around 90% after a four-day period. Figure (b) shows the number of surviving cells in time, normalized by the initial measurement after two hours. For both sub-figures, the error bars indicate the standard deviations of cell counts from different areas in the same well.

References

[1] C. Clanet, C. Béguin, D. Richard, and D. Quéré, “Maximal deformation of an impacting drop,” *Journal of Fluid Mechanics*, vol. 517, pp. 199–208, Sept. 2004.

[2] S. Tasoglu, G. Kaynak, A. J. Szeri, U. Demirci, and M. Muradoglu, “Impact of a compound droplet on a flat surface: A model for single cell epitaxy.,” *Physics of Fluids*, vol. 22, pp. 1–15, Aug. 2010.

[3] C. W. Visser, P. E. Frommhold, S. Wildeman, R. Mettin, D. Lohse, and C. Sun, “Dynamics of high-speed micro-drop impact: numerical simulations and experiments at frame-to-frame times below 100 ns,” *Soft Matter*, vol. 11, pp. 1708–1722, Dec. 2015.

[4] Pharmacosmos, “http://www.dextran.net/about-dextran/dextran-chemistry/physical-properties.aspx,” 2014.
